# Analysis and Construction of a Molecular Diagnosis Model of Drug-Resistant Epilepsy Based on Bioinformatics

**DOI:** 10.3389/fmolb.2021.683032

**Published:** 2021-11-05

**Authors:** Tenghui Han, Zhenyu Wu, Jun Zhu, Yao Kou, Jipeng Li, Yanchun Deng

**Affiliations:** ^1^ Department of Neurology, Xijing Hospital, Airforce Medical University, Xi’an, China; ^2^ Department of Anatomy, Histology and Embryology and K.K. Leung Brain Research Centre, School of Basic Medicine, Airforce Medical University, Xi’an, China; ^3^ State Key Laboratory of Cancer Biology, Institute of Digestive Diseases, Xijing Hospital, Airforce Medical University, Xi’an, China; ^4^ Department of General Surgery, The Southern Theater Air Force Hospital, Guangzhou, China; ^5^ Basic Medical College, Yan’an University, Yan’an, China

**Keywords:** epilepsy, drug-resistant epilepsy, bioinformatics analysis, CREB signaling pathway, resistance gene

## Abstract

**Background:** Epilepsy is a complex chronic disease of the nervous system which influences the health of approximately 70 million patients worldwide. In the past few decades, despite the development of novel antiepileptic drugs, around one-third of patients with epilepsy have developed drug-resistant epilepsy. We performed a bioinformatic analysis to explore the underlying diagnostic markers and mechanisms of drug-resistant epilepsy.

**Methods:** Weighted correlation network analysis (WGCNA) was applied to genes in epilepsy samples downloaded from the Gene Expression Omnibus database to determine key modules. The least absolute shrinkage and selection operator (LASSO) regression and support vector machine-recursive feature elimination (SVM-RFE) algorithms were used to screen the genes resistant to carbamazepine, phenytoin, and valproate, and sensitivity of the three-class classification SVM model was verified through the receiver operator characteristic (ROC) curve. A protein–protein interaction (PPI) network was utilized to analyze the protein interaction relationship. Finally, ingenuity pathway analysis (IPA) was adopted to conduct disease and function pathway and network analysis.

**Results:** Through WGCNA, 72 genes stood out from the key modules related to drug resistance and were identified as candidate resistance genes. Intersection analysis of the results of the LASSO and SVM-RFE algorithms selected 11, 4, and 5 drug-resistant genes for carbamazepine, phenytoin, and valproate, respectively. Subsequent union analysis obtained 17 hub resistance genes to construct a three-class classification SVM model. ROC showed that the model could accurately predict patient resistance. Expression of 17 hub resistance genes in healthy subjects and patients was significantly different. The PPI showed that there are six resistance genes (*CD247*, *CTSW*, *IL2RB*, *MATK*, *NKG7*, and *PRF1*) that may play a central role in the resistance of epilepsy patients. Finally, IPA revealed that resistance genes (*PRKCH* and *S1PR5*) were involved in “CREB signaling in Neurons.”

**Conclusion:** We obtained a three-class SVM model that can accurately predict the drug resistance of patients with epilepsy, which provides a new theoretical basis for research and treatment in the field of drug-resistant epilepsy. Moreover, resistance genes *PRKCH* and *S1PR5* may cooperate with other resistance genes to exhibit resistance effects by regulation of the cAMP-response element-binding protein (CREB) signaling pathway.

## 1 Introduction

Epilepsy is a complex chronic neurological disease characterized by the recurrence of unprovoked seizures and has numerous neurobiological, cognitive, and psychosocial consequences (Fisher et al., 2014). It affects the health of over 70 million people worldwide (Thijs et al., 2019). Epilepsy has complex etiologies, diverse clinical symptoms and phenotypes, and high heterogeneity, which interfere with its diagnosis as well as treatment (Rawat et al., 2020). Moreover, approximately a third of patients with epilepsy are refractory to antiepileptic drugs (AEDs) when they are employed singly or even in various combinations (Lerche, 2020). There is thus an urgent need to find new diagnostic markers of refractory epilepsy to ameliorate the current situation of epilepsy diagnosis and treatment.

There are multitypes of AEDs for epilepsy treatment, among which carbamazepine (CBZ), phenytoin (PHT), and valproate (VPA) are the most widely used first-line drugs (Schmidt and Schachter, 2014). CBZ is a first-line treatment for partial and generalized convulsive seizures, trigeminal pain, and bipolar disorder, which functions as a Na^+^ channel blocker (Harper and Topol, 2012). CBZ remains the most efficacious drug for focal and generalized seizures with focal onset (Baulac et al., 2012; Baulac et al., 2017). PHT is also speculated to work as a Na^+^ channel blocker; it exhibits similar efficacy to CBZ and is the first-line drug for focal seizures and generalized seizures with focal onset. Unusually, PHT is mainly administered intravenously (Mattson et al., 1985). As the first-line and most effective intravenous drug for focal and generalized seizures in current clinical treatment, VPA performs multiple functions, including GABA potentiation, glutamate inhibition, and sodium channel and T-type calcium channel blockade (Tomson et al., 2016).

In 2009, the International League Against Epilepsy (ILAE) defined drug-resistant epilepsy as “failure of adequate trials of two tolerated, appropriately chosen and used AED schedules” (Kwan et al., 2010). Patients with drug-resistant epilepsy have a significantly increased risk of psychiatric and somatic comorbidities and adverse effects from AEDs. Furthermore, their seizures are not well controlled and recurrent, especially generally tonic–clonic seizures, which is the best-recognized risk factor for sudden unexplained death in epilepsy (Ryvlin et al., 2019). Recent research has demonstrated that after the failure of two well-tolerated AED schedules appropriately chosen for the seizure types, patients under long-term treatment for epilepsy have a progressively less likely chance of success with further drug treatment (Chen et al., 2018). Therefore, early-stage identification of AED resistance is crucial to patient treatment outcomes.

**FIGURE 1:**
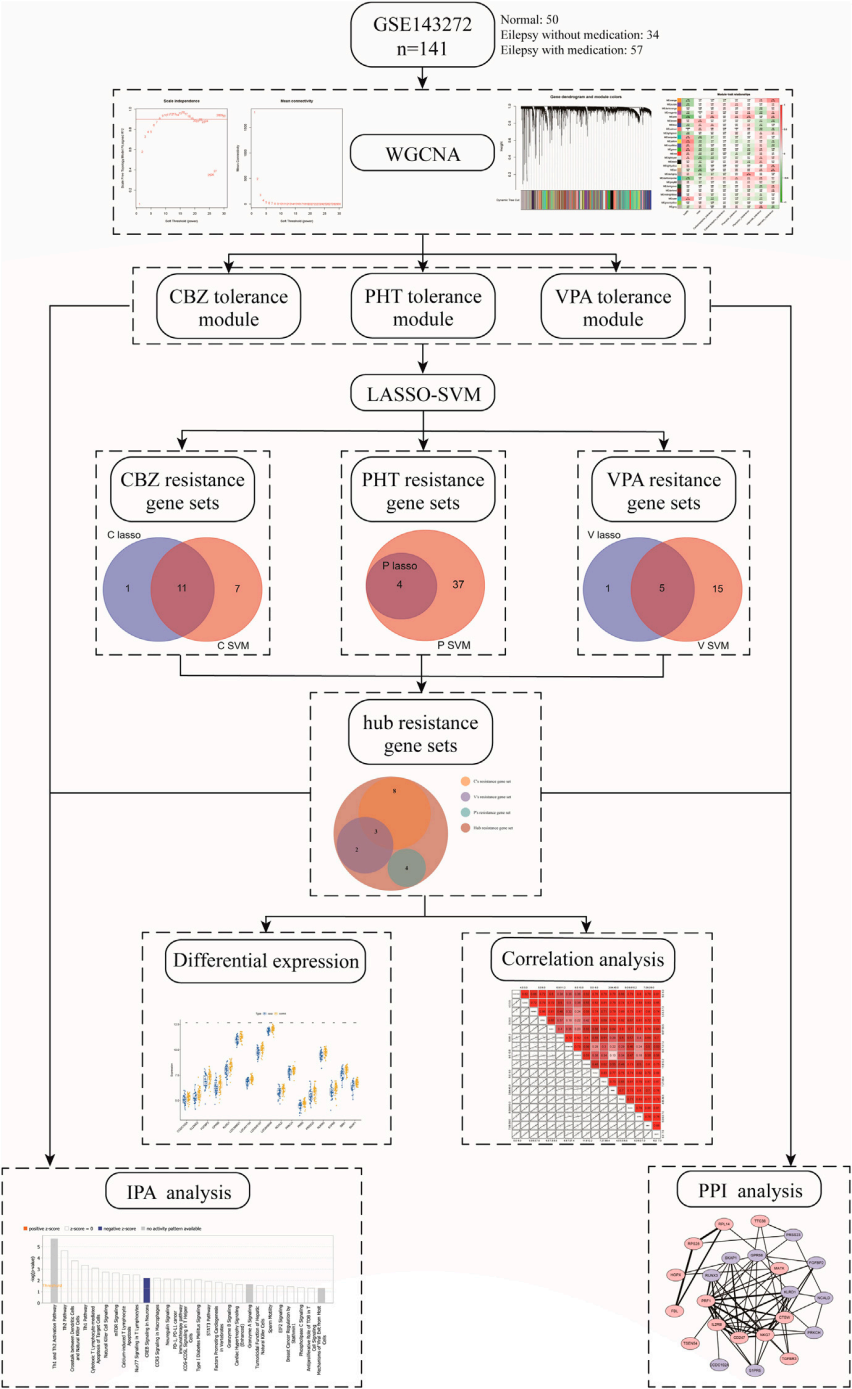
Workflow of the study.

In our study, we used weighted correlation network analysis (WGCNA), the least absolute shrinkage and selection operator (LASSO) algorithm, and the support vector machine-recursive feature elimination (SVM-RFE) algorithm to analyze and select resistance genes. All genes in epilepsy patient samples were downloaded from the Gene Expression Omnibus (GEO) database. We constructed a novel three-class classification SVM model to accurately predict patient resistance, which may provide a new strategy for the treatment and research of drug-resistant epilepsy and also revealed that the resistance genes *PRKCH* and *S1PR5* may cooperate with other resistance genes through regulation of the cAMP-response element-binding protein (CREB) signaling pathway. The workflow is shown in Figure 1.

## 2 Materials and Methods

### 2.1 Data Source

The original dataset of the whole gene expression profiles was downloaded from the GEO database. The accession number was GSE143272, which was based on GPL10558 (Illumina HumanHT-12 V4.0 expression beadchip). Gene sequences of a total of 34 drug-naïve patients with epilepsy and 57 followed-up patients showing differential response to AED monotherapy, along with 50 healthy subjects as a control group, were included in the study. The AED-treatment group included the CBZ-drug-treatment group (tolerance: 9; intolerance: 10), the PHT-drug-treatment group (tolerance: 6; intolerance: 7), and the VPA-drug-treatment group (tolerance: 9; intolerance: 16).

### 2.2 Definitions of Candidate Resistance Genes by WGCNA

In this study, we used the “WGCNA” R software package to construct modules related to clinical features in the epilepsy sample dataset (GSE143272) and identify candidate genes (Langfelder and Horvath, 2008). The clinical features were divided into eight categories: normal (health), unmedicated epilepsy (case), CBZ tolerance, CBZ intolerance, PHT tolerance, PHT intolerance, VPA tolerance, and VPA intolerance. The overall clustering of the GSE143272 dataset was found to be of relatively high quality, so no sample removal processing was performed (Supplementary Figure S1A). The traits of the samples are shown in Supplementary Figure S1B. The adjacency matrix was converted to a topological overlap matrix (TOM) (Li et al., 2019). According to the degree of TOM similarity, genes were divided into multiple gene modules (Supplementary Figures S1C,D). In this analysis, the soft threshold was set to 7 (scale-free R^2^ = 0.85), and the minimum module size was 30. The correlations between the characteristic gene of each module and clinical characteristics were calculated. The screening of key modules was achieved by calculating the correlation between the module genes and clinical features. Moreover, a gene with |gene significance (GS)| >0.2 and |module membership (MM)| > 0.8 in the key modules was considered as a candidate resistance gene.

### 2.3 Feature Selections by LASSO and SVM-RFE Algorithms

LASSO logistic regression and SVM-RFE were performed on the candidate resistance genes obtained in WGCNA to screen characteristic genes. LASSO is a regression analysis algorithm that uses regularization to improve the prediction accuracy. The penalty parameter (λ) of the LASSO regression model was determined by following a 10-fold cross-validation of the minimum criterion (i.e., the value of λ corresponding to the lowest partial likelihood deviation). The LASSO regression algorithm using the “glmnet” package (Friedman et al., 2010) in R was performed to identify genes significantly associated with the distinctions between CBZ-resistant and PHT + VPA-resistant samples, PHT-resistant and CBZ + VPA-resistant samples, and VPA-resistant and CBZ + PHT-resistant samples. Furthermore, SVM-RFE is an effective feature selection technique that finds the best variables by deleting the feature vector generated by SVM (Wang and Liu, 2015). In this study, the SVM-RFE algorithm screened the best variables based on a minimum 10 × CV error value. The performances of CBZ/PHT/VPA resistance LASSO and SVM models are shown in Supplementary Table S1. For each drug, resistance genes were defined as the common genes identified by the LASSO and SVM-RFE algorithms. Ultimately, we combined the resistance genes of CBZ, PHT, and VPA as hub resistance genes for further analysis. A three-class classification SVM module was established using the “e1071” software package in R (Supplementary Figure S2) (Cinelli et al., 2017), and the receiver operating characteristic (ROC) curve was used to further determine the diagnostic value of the hub resistance genes in epilepsy.

### 2.4 Construction of the Protein–Protein Interaction Network

To interpret the molecular mechanisms of hub resistance genes in epilepsy, the online tool, the Search Tool for the Retrieval of Interacting Genes (STRING) database, was used to construct the protein–protein interaction (PPI) network of 72 modular genes (Szklarczyk et al., 2015). The PPI was visualized with a confidence score >0.15 (Assenov et al., 2008).

### 2.5 Ingenuity Pathway Analysis for the Identification of Diseases and Function Pathways Involved

Ingenuity pathway analysis (IPA) is a web-based bioinformatic application for functional analysis, aggregation, and further understanding of data analysis results (Khan et al., 2016). Briefly, IPA was performed to identify diseases and functions and gene networks that were most significant to hub resistance genes. The Z-scores of significantly involved diseases and function pathways were also determined.

### 2.6 Statistical Analysis

All statistical analyses were performed using R version 3.4.1. The Wilcox test was used to analyze the relationship between drug resistance and clinicopathological characteristics. Pearson correlation analysis was adopted to understand the relevance of the 17 hub resistance genes. The area under the curve (AUC) was calculated to evaluate the property of the models. *p* < 0.05 was envisaged to indicate a statistically significant difference.

## 3 Results

### 3.1 Determination of the Most Relevant Module Genes for Drug Tolerance in Epilepsy Treatment

**FIGURE 2:**
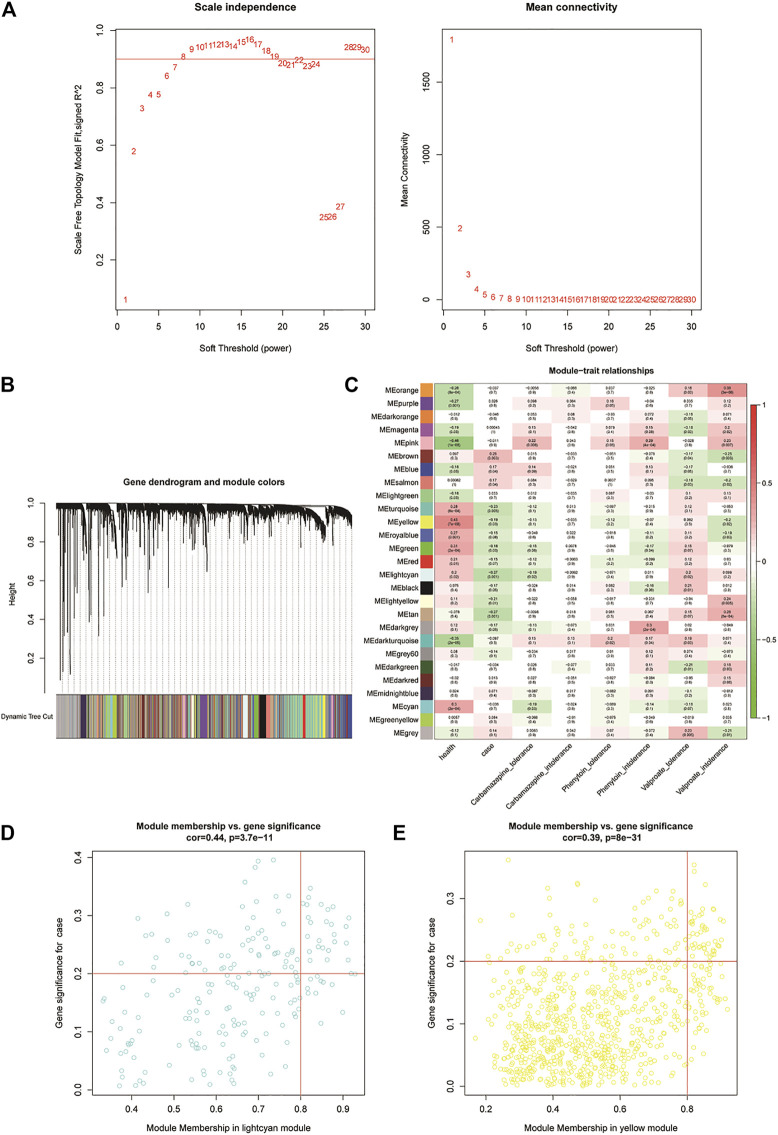
Weighted gene co-expression network analysis of the potential resistance genes. **(A)** Weighted value β of scale-free networks. The relationship between the soft threshold and scale-free R2 is exhibited on the left. On the right, the relationship between the soft threshold and mean connectivity is shown. **(B)** Cluster dendrogram. Each branch in the figure represents the genes, which are divided into module colors based on the cluster analysis results. The oligogenics are assigned in the gray module. **(C)** Heatmap of the correlation analysis between modules and clinical characteristics. The vertical axis represents the different modules; the horizontal axis represents the different traits. The number in each cell represents the correlation coefficient and significance (*p*-value) between a module and a trait. **(D,E)** Scatter diagrams of MElightcyan and MEyellow modules. Using the criteria |GS| > 0.2 and |MM| > 0.8, we selected the key genes of each module in the upper right corner of the figure. Twenty-five key genes were screened from the MElightcyan module, a resistance module common to both CBZ and VPA drugs.

We first clustered all the samples in the GSE143272 dataset to ensure the accuracy of the analysis (Supplementary Figure S1A). The coexpression network was constructed through coexpression analysis. A total of 27 modules (including gray modules) were identified via the average linkage hierarchical clustering. To ensure that the interaction between genes in the coexpression network could conform to the scale-free distribution to the greatest extent, the power of *β* = 7 was selected; to merge the highly similar modules, we chose a cutoff <0.25 and a minimum module size of 30 using the dynamic hybrid tree cut method. In this study, we focused on the drug-resistant traits of disease samples. Therefore, we included the two traits of the case and drug tolerance as reference factors to screen key modules. It was found that the MElightcyan module had the highest correlation with CBZ-tolerance traits (module-trait relationships = −0.27 and −0.12, respectively) and VPA-tolerance traits (module-trait relationships = −0.27 and 0.2, respectively) of cases. The MEyellow module (module-trait relationships = −0.19 and −0.12, respectively) was found to have the highest association with the PHT-tolerance status of the case (Figure 2). Hence, 1,016 genes in the two modules (MElightcyan: 206 and MEyellow: 810) were considered to be significant module genes for further intramodular analysis. Based on the candidate gene screening criteria in the key module (|GS| > 0.2 and |MM| > 0.8), a total of 72 candidate genes from the MElightcyan (25 genes) and MEyellow (47 genes) modules were chosen for further analysis (Figures 2E,F; Supplementary Tables S2, S3).

### 3.2 Identification of Hub Resistance Genes in Patients With Epilepsy

In this study, two distinct algorithms, LASSO and SVM-RFE, were utilized for screening potential resistance genes against CBZ, PHT, and VPA. For each drug, resistance genes were defined by the common signature genes identified by LASSO and SVM-RFE. Ultimately, the resistance genes of all three drugs were collectively termed as hub resistance genes in our research.

**FIGURE 3:**
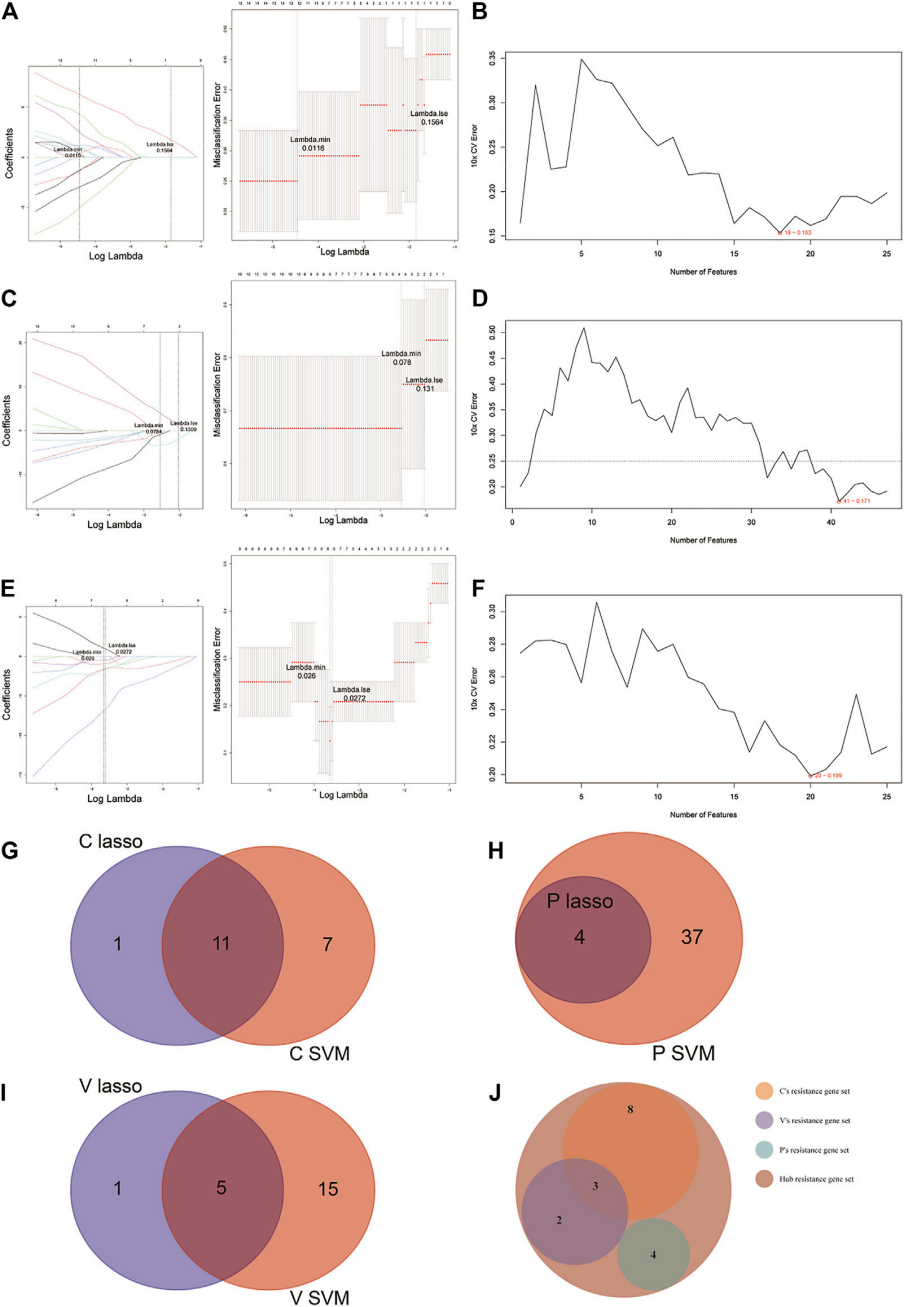
LASSO and SVM-RFE algorithms were used for characteristic gene selection. **(A, C, E)** LASSO algorithm. Using the LASSO algorithm, we identified 12 potential resistance genes in the CBZ-resistance gene set, 4 in the PHT-resistance gene set, and 6 in the VPA-resistance gene set. **(B, D, F)** SVM-RFE algorithm. SVM-RFE algorithm separately indicated the resistance genes most closely corresponding with the lowest error rates in patients treated with CBZ **(B)**, PHT **(D)**, and VPA **(F)**. **(G–I)** Venn diagram of the characteristic genes for CBZ **(G)**, PHT **(H)**, and VPA **(I)**, which were selected from the LASSO or SVM-RFE algorithms. **(J)** We unified the LASSO + SVM characteristic resistance genes of CBZ, PHT, and VPA and obtained 17 characteristic genes.

For the identification of potential resistance genes to CBZ, we built classifiers capable of distinguishing between CBZ-resistant samples (*n* = 9) and PHT + VPA-resistant samples (*n* = 15) using the LASSO and SVM-RFE algorithms. Specifically, the LASSO regression was performed to remove candidate genes that were related to each other to prevent overfitting of the model (Figure 3A). A total of 12 LASSO signature genes were obtained at *λ* min = 0.0116; they were *CCDC102A*, *CEP78, CLDND2*, *FGFBP2*, *GPR56*, *KLRD1*, *NCALD*, *PRKCH*, *RUNX3*, *S1PR5*, *SBK1*, and *SKAP1*. Meanwhile, based on the SVM-RFE algorithm (Figure 3B), 18 SVM-RFE signature genes were identified at a minimum 10-fold CV error (0.153), namely, *NCALD*, *FGFBP2*, *CCDC102A*, *KLRD1*, *S1PR5*, *SKAP1*, *TTC38*, *CLDND2*, *PRKCH*, *SBK1*, *CD247*, *RUNX3*, *ENPP4*, *TSEN54*, *NKG7*, *PRR5*, *GPR56*, and *HOPX*. Subsequently, a total of 11 genes (*CCDC102A*, *CLDND2*, *FGFBP2*, *GPR56*, *KLRD1*, *NCALD*, *PRKCH*, *RUNX3*, *S1PR5*, *SBK1*, and *SKAP1*) were identified by overlap analysis as common to both the LASSO signature gene set and the SVM-RFE signature gene set; these genes were defined as resistance genes for CBZ (Figure 3G).

Before identifying potential resistance genes to PHT, we divided all drug-resistant samples into PHT-resistant (*n* = 6) and CBZ + VPA-resistant (*n* = 18) groups. The 72 candidate genes previously identified were narrowed down using the LASSO regression algorithm, resulting in the identification of four variables (*LOC388621*, *LOC441154*, *LOC645157*, and *LOC649548*) as potential resistance genes for PHT at λ min = 0.0784 (Figure 3C). Based on the best point (10 × CV error = 0.171), the SVM-RFE algorithm obtained 41 eigenvalues (Figure 3D; Supplementary Table S4). By overlapping the genes from the two algorithms, we identified the four genes (*LOC388621*, *LOC441154*, *LOC645157*, and *LOC649548*) as resistance genes in patients treated with PHT (Figure 3H).

Based on 9 VPA-resistant samples and 15 CBZ + PHT-resistant samples, the LASSO regression algorithm identified *IL2RB*, *NCALD, PRKCH*, *PRR5*, *PRSS23*, and *RUNX3* as potential resistance genes to VPA based on *λ* min = 0.0272 from 72 candidate genes (Figure 3E). A subset of 16 features among the candidate genes was determined using the SVM-RFE algorithm (10 × CV error = 0.199; Figure 3F). The five overlapping features (*NCALD*, *PRKCH*, *PRR5*, *PRSS23*, and *RUNX3*) between these two algorithms were ultimately selected as the resistance genes in patients treated with VPA (Figure 3I).

Collectively, we obtained a total of 11 CBZ-resistant genes, 4 PHT-resistant genes, and 5 VPA-resistant genes (Supplementary Table S5). Overlap analysis revealed that *NCALD*, *RUNX3*, and *PRKCH* were the common resistance genes for CBZ and VPA (Figure 3J). Thus, a total of 17 hub resistance genes were obtained and included for further analysis.

### 3.3 Evaluation of the Three-Class Classification SVM Model

**FIGURE 4:**
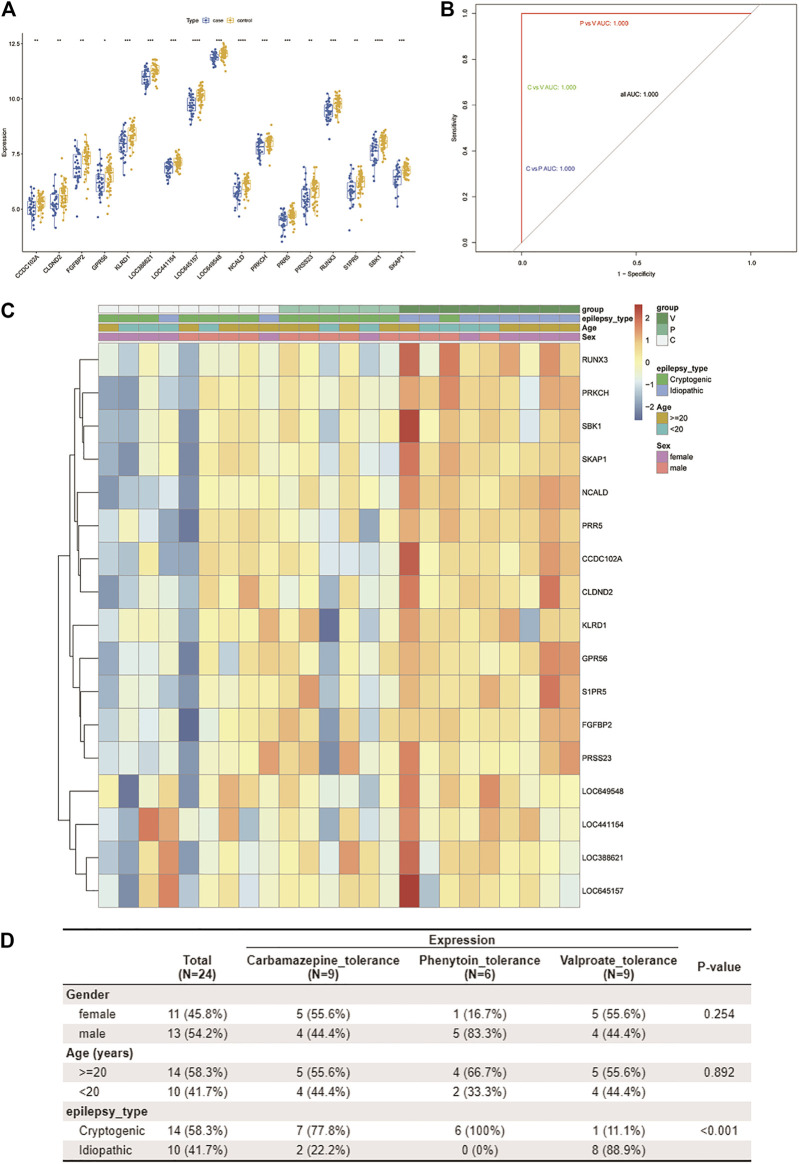
Assessment of the predictive value of the three-class classification SVM model. **(A)** Boxplot shows the expression patterns of 17 drug resistance genes in case and control samples from the GSE143272 dataset. ^*^ *p* < 0.05, ^**^ *p* < 0.01, ^***^ *p* < 0.001, and ^****^ *p* < 0.0001. **(B)** ROC curve based on every two drugs in the model. Blue represents CBZ vs. PHT, green represents CBZ vs. VPA, and red represents PHT vs. VPA. Since all AUCs are 1.000, only one color is shown in the figure (other ROC curves are covered). **(C,D)** By using the Wilcox test, we analyzed the correlation between three clinical traits (age, gender, and pathological classification) and drug resistance. A heatmap of resistance genes and clinical traits is plotted **(C)**. Clinical traits and drug resistance were significantly correlated (*p* < 0.05) **(D)**.

The 17 resistance genes were significantly different in control and case samples; i.e., compared with the control group, their expression in case samples was generally lower (Figure 4A). Then, the library (“e1071”) package was used in the R software to construct a three-class classification SVM model for the 17 hub resistance genes obtained from the above analysis, and its prediction performance was evaluated in the GSE143272 dataset. The ROC curve was drawn based on the true and predicted values of each two drugs in the model. The results demonstrated that the three-class classification SVM model could distinguish the patient’s tolerance to the three drugs (all AUC = 1.000), indicating that the resistance genes may be clinically useful (Figure 4B). We then compared the clinical characteristics of the three subgroups, namely, CBZ tolerance, PHT tolerance, and VPA tolerance. Subgroup analysis of clinical characteristics showed that the cryptogenic epilepsy type was characterized by significant differences (Figures 4C,D). Other clinical characteristics like gender, age, and idiopathic epilepsy type had no statistical significance.

### 3.4 Correlation Analysis of Resistance Genes

**FIGURE 5:**
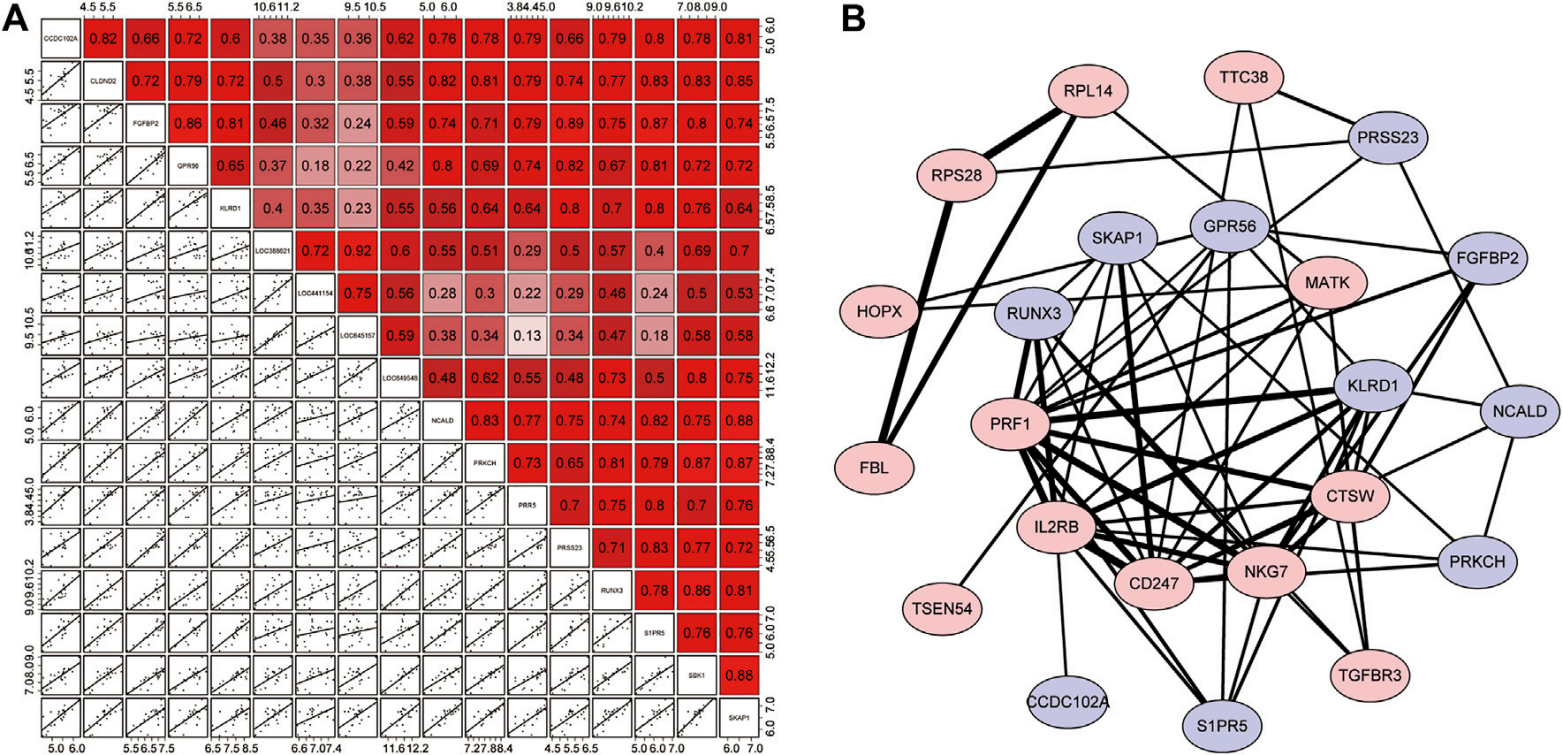
Correlation analysis of resistance genes and the PPI network. **(A)** Correlation matrix of 17 resistance genes. The upper right half is the correlation score, where red represents positive correlation and purple indicates negative correlation. The lower left section shows scattered plots of correlations between each of the two genes. **(B)** PPI network. The red proteins are resistance genes, while the blue proteins are the remaining module genes. Based on the combined score, the lines represented the interaction between them, and the degree of thickness represented the degree of their combination.

Pearson analysis was used to explore the correlation between 17 resistance genes. Studies have shown that all resistance genes have a strong positive correlation; as shown in Figure 5A, *SKAP1* has the highest correlation with *SBK1* and *NCALD* (*r* = 0.88). The relationship between some other resistance genes does not seem to be as close. For example, the correlation between *LOC645157* and *PRR5*/*S1PR5* (*r* = 0.13 and *r* = 0.18, respectively) and the correlation between *GPR56* and *LOC441154* were not considerable (*r* = 0.18).

Next, we used the STRING online tool to construct a PPI network for 72 modular genes. This was to show the maximum possible additional modular genes that interacted with the 17 resistance genes. We set the confidence level to 0.15. After removing discrete proteins, we obtained a PPI network with 23 proteins. The PPI network is illustrated in Figure 5B. The results showed that 6 of the 17 resistance genes were at the center of the network, indicating that they were associated with a higher number of genes. Therefore, we speculated that these genes played a major role in the corresponding drug-tolerance modules. Judging from the analysis of the degree of binding (combined score), we found that *CD247*-*IL3RB*-*PRF1*-*NKG7*/*KLRD1*/*CD274* may form a complete closed loop of tolerance and promote the patient’s body to develop resistance. Also, although *RPL14*, *RPS28*, and *FBL* were out of the core of the PPI, these three resistance genes could form a complete closed chain of action and exert a powerful resistance effect (Supplementary Table S6). Regardless of the fact that only 10 resistance genes were displayed in the network, the remaining 7 resistance genes seem to have a unique relationship network that was not yet known to play their corresponding roles.

### 3.5 IPA of the Hub Resistance Genes

**FIGURE 6:**
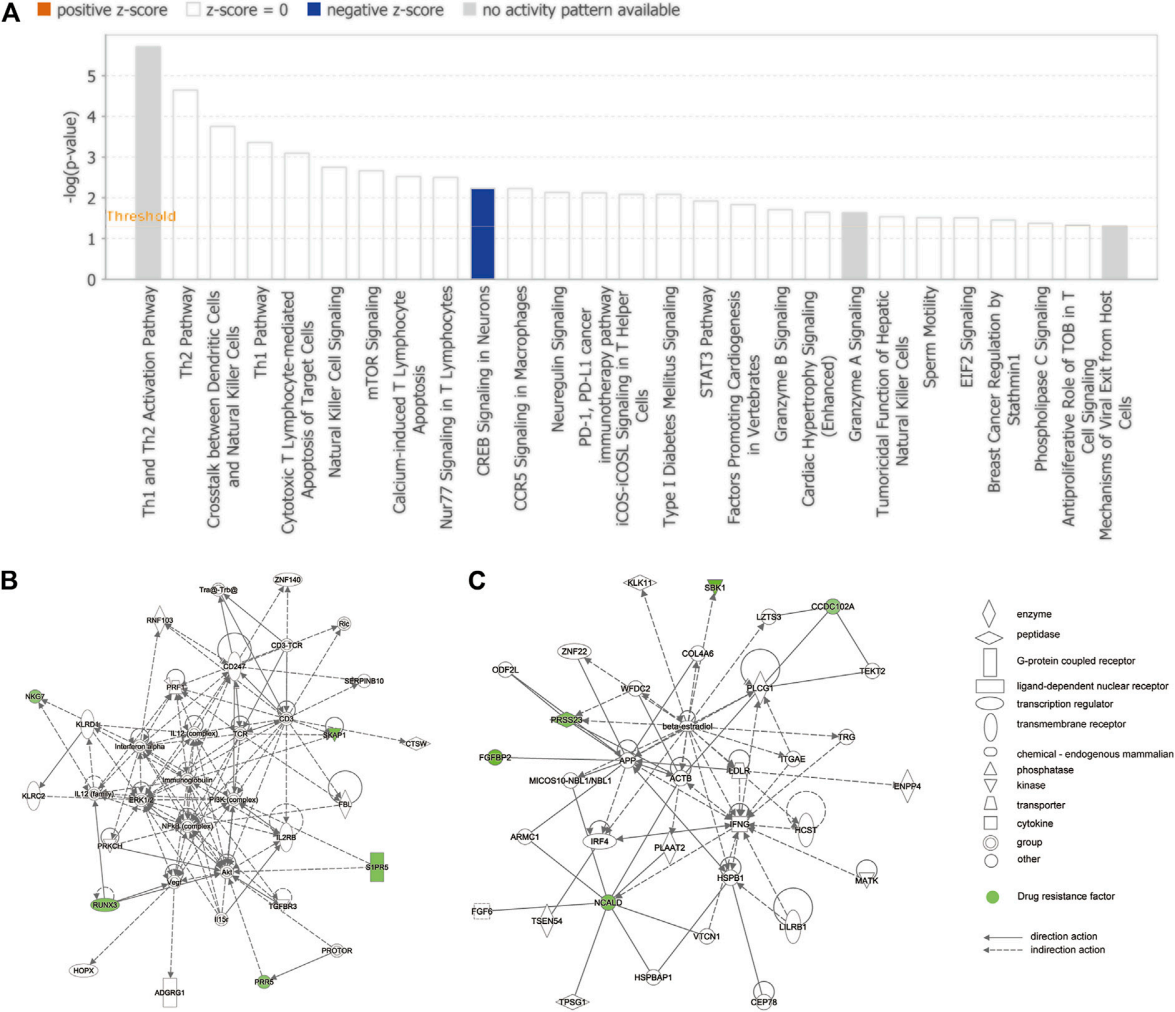
IPA of module genes. **(A)** Diseases and functional pathway analysis of module genes. While the Y-axis is the pathway terms, the X-axis is the log (*p*-value). **(B)** Interaction network was constructed between modules of genes and the chemical/drug and other substances by using IPA. Green represents resistance genes, including *KLRD1*, *PRR5*, *RUNX3*, *S1PR5*, *SKAP1*, *CCDC102A*, *FGFBP2*, *NCALD*, *PRSS23*, and *SBK1*. Solid lines of the arrows indicate direct interactions between genes, while dotted lines indicate indirect interactions.

The complete list of enriched disease and function pathway analysis is included in Supplementary Table S7. A total of 27 enriched disease and function pathways were identified by applying the −log (*p*-value) > 1.3 threshold. All the 27 representative pathways that were found to associate tightly with the tolerance module genes and resistance genes are shown in Figure 6A, ranked according to their −log (*p*-value). The “Th1 and Th2 activation pathway” was the highest-ranking signaling pathway with a −log (*p*-value) of 5.71. Although none of the detected signaling pathways had a Z-score > 2 (significant activation), one of the enriched signaling pathways, “CREB signaling in neurons,” had a Z-score = −2. Of note, the involvement of CREB in the occurrence and development of epilepsy is well recognized (Sharma et al., 2019). These results suggest that these resistance genes (*PRKCH* and *S1PR5*) may induce resistance in patients with drug-treated epilepsy by regulation of the CREB pathway. Moreover, Figure 6B shows the interaction network between 72 modular genes. Among them, we found that *RUNX3* could directly interact with *S1PR5* and *PPR5* by acting on *Akt*. However, *CCD102A*, *FGFBP2*, *NCALD*, *PRSS23*, and *SRSS23* were intertwined into an intricate network through their direct or indirect interaction with beta-estradiol.

## 4 Discussion

Epilepsy is one of the most common chronic diseases of the nervous system and extensively affects people of all ages, genders, and races worldwide (Fiest et al., 2017; Devinsky et al., 2018). Pharmacological treatment is widely recognized as the mainstay of the therapy approach for people with epilepsy. However, previous studies have indicated that more than one-third of the patients are likely to develop refractory epilepsy in the process of AED treatment (Kwan and Brodie, 2000; Löscher et al., 2020). The complex resistance mechanisms of AEDs are still not entirely clear. Recent studies have demonstrated that the application of bioinformatic analysis could provide a chance to explore the underlying mechanisms of drug resistance (Zhang et al., 2019; Zhu et al., 2019). Therefore, we utilized bioinformatic analysis techniques to construct a three-class SVM model to precisely predict the drug resistance of patients with epilepsy and explored the potential mechanisms of drug-resistant epilepsy.

In this study, we included 50 healthy patients, 34 patients with epilepsy untreated by medication, and 57 patients with epilepsy with three different AED treatments (CBZ, PHT, or VPA) from the GEO database (GSE143272 dataset). Then, 72 candidate resistance genes were identified by WGCNA. IPA revealed a total of 27 disease and functional associations of candidate resistance genes. The highest-ranked signaling pathway was the Th1 and Th2 activation pathway, indicating that candidate resistance genes were potentially involved in the regulation of immune response in patients. Subsequently, by employing the LASSO + SVM-RFE algorithm, we constructed a three-class classification SVM model based on 17 hub resistance genes (*CCDC102A*, *CLDND2*, *FGFBP2*, *GPR56*, *KLRD1*, *NCALD*, *PRKCH*, *PRR5*, *PRSS23*, *RUNX3*, *S1PR5*, *SBK1*, *SKAP1*, *LOC388621*, *LOC441154*, *LOC645157*, and *LOC649548*) from CBZ-resistant, PHT-resistant, and VPA-resistant gene sets. The model possessed a strong ability to predict drug tolerance in patients (AUC = 1.000). Furthermore, these genes displayed a significant Pearson correlation with each other. The PPI network analysis revealed that *CD247*, *CTSW*, *IL2RB*, *MATK*, *NKG7*, and *PRF1* were at the center of the network and may play essential roles in the development of drug resistance.

Our study screened 17 novel resistance genes and built a highly effective model to accurately predict the drug resistance of patients with epilepsy. Among the 17 hub resistance genes, we found that *NCALD* and *GPR56* were verified to be directly relevant to epilepsy in previous studies. Recent studies have reported that intellectual disability and epilepsy were detected in patients with *NCALD* deletion, indicating that *NCALD* could be a crucial gene in epilepsy (Kuechler et al., 2011; Kuroda et al., 2014). Additionally, studies have demonstrated that *GPR56* mutations may cause malformations of cortical development, which could further result in epileptogenesis (Guerrini and Dobyns, 2014; Kuzniecky, 2015). However, the underlying mechanisms of *NCALD* and *GPR56* in AED resistance have not yet been reported and left a wide scope for further research.

Considering the above result of the IPA, we found that the CREB signaling pathway in neurons appeared to be closely associated with the tolerance module and resistance genes. Recent research has demonstrated that the CREB signaling pathway plays an essential role in mossy fiber sprouting, which is generally known to be a pathological result of recurrent epilepsy. CREB upregulation boosts the transcription of its target genes, which results in the enhancement of mossy fiber sprouting and an increase in the number of dysfunctional synapses in neural circuits, resulting in poor AED treatment outcomes for patients with epilepsy and ultimately developing into refractory epilepsy (Redmond et al., 2002; Finsterwald et al., 2010). Additionally, according to our results, two hub resistance genes (*PRKCH* and *S1PR5*) were closely involved in the CREB pathway, which is consistent with previous research. *PRKCH* encodes a protein kinase subtype, which is widely involved in brain functions (Boehm et al., 2006; Schwenk et al., 2013). Through pathway analysis on the identified single-nucleotide polymorphism component, researchers have found that *PRKCH* is strongly associated with the CREB signaling pathway (Chen et al., 2015). *S1PR5* encodes a G-protein-coupled receptor which is reported to be highly relevant to CREB activation (Rivera et al., 2008; Wang et al., 2020). Moreover, *PRKCH* was proved to be the joint gene among CBZ-resistant and VPA-resistant gene sets in our findings. Integrating this evidence, we speculate that *PRKCH* and *S1PR5* may induce resistance in patients with drug-treated epilepsy through the CREB pathway.

Intriguingly, emerging evidence has demonstrated that *PRKCH* and *PPR5* are associated with the mTOR signaling pathway. The mTOR pathway regulates a variety of neuronal functions, including cell proliferation, survival, growth, metabolism, and plasticity. Compelling evidence has indicated that abnormal activity of the mTOR pathway plays an irreplaceable role in epileptogenesis (Lim et al., 2015; Curatolo et al., 2018). Moreover, recent studies have further confirmed the substantial therapeutic potential of targeting the mTOR signaling pathway in drug-resistant epilepsy (Hodges and Lugo, 2020). This implies that *PRKCH* and *PPR5* could be potential targets for the treatment of refractory epilepsy.

Additionally, other than the 5 hub genes mentioned above, we also identified 12 novel drug resistance genes, herein first reported to be related to refractory epilepsy. According to the correlation analysis, all 17 resistance genes have a strong positive relation, and *SKAP1* has the highest correlation, with *SBK1* and *NCALD*. Moreover, among the 12 novel resistance genes, *CCDC102A*, *FGFBP2*, *RUNX3*, *SKAP1*, *KLRD1*, and *PRSS23* were intertwined into a complex PPI network. *LOC388621*, *LOC441154*, *LOC645157*, and *LOC649548* were first screened out to be PHT-resistant genes, and their structure and function deserve to be further studied. Integrating the results above, we inferred that the 17 hub genes have intricate direct or indirect interactions in drug-resistant epilepsy.

Nevertheless, there were several limitations in this study. First, our research is based on a publicly available dataset. Prospective real-world data should be incorporated to validate the clinical utility of our model. Subsequently, further *in vitro* and *in vivo* experiments should be performed to confirm the mechanisms of the 17 hub genes in drug-resistant epilepsy.

## 5 Conclusion

Through this study, we have offered novel insights into the research and treatment of drug-resistant epilepsy and created a novel three-class SVM model with high prediction values. This is also the first study that has elucidated that the resistance genes *PRKCH* and *S1PR5* may work in coordination with other resistance genes to exhibit their resistance effects through regulation of the CREB signaling pathway.

## Data Availability

The datasets presented in this study can be found in online repositories. The names of the repository/repositories and accession number(s) can be found in the article/Supplementary Material.

## References

[B1] Assenov Y. Ramírez F. Schelhorn S.-E. Lengauer T. Albrecht M. (2008). Computing Topological Parameters of Biological Networks. Bioinformatics 24 (2), 282–284. 10.1093/bioinformatics/btm554 18006545

[B2] Baulac M. Brodie M. J. Patten A. Segieth J. Giorgi L. (2012). Efficacy and Tolerability of Zonisamide versus Controlled-Release Carbamazepine for Newly Diagnosed Partial Epilepsy: a Phase 3, Randomised, Double-Blind, Non-inferiority Trial. Lancet Neurol. 11 (7), 579–588. 10.1016/s1474-4422(12)70105-9 22683226

[B3] Baulac M. Rosenow F. Toledo M. Terada K. Li T. De Backer M. (2017). Efficacy, Safety, and Tolerability of Lacosamide Monotherapy versus Controlled-Release Carbamazepine in Patients with Newly Diagnosed Epilepsy: a Phase 3, Randomised, Double-Blind, Non-inferiority Trial. Lancet Neurol. 16 (1), 43–54. 10.1016/s1474-4422(16)30292-7 27889312

[B4] Boehm J. Kang M.-G. Johnson R. C. Esteban J. Huganir R. L. Malinow R. (2006). Synaptic Incorporation of AMPA Receptors during LTP Is Controlled by a PKC Phosphorylation Site on GluR1. Neuron 51 (2), 213–225. 10.1016/j.neuron.2006.06.013 16846856

[B5] Chen J. Calhoun V. D. Arias-Vasquez A. Zwiers M. P. van Hulzen K. Fernández G. (2015). G-protein Genomic Association with normal Variation in gray Matter Density. Hum. Brain Mapp. 36 (11), 4272–4286. 10.1002/hbm.22916 26248772 PMC5667539

[B6] Chen Z. Brodie M. J. Liew D. Kwan P. (2018). Treatment Outcomes in Patients with Newly Diagnosed Epilepsy Treated with Established and New Antiepileptic Drugs. JAMA Neurol. 75 (3), 279–286. 10.1001/jamaneurol.2017.3949 29279892 PMC5885858

[B7] Cinelli M. Sun Y. Best K. Heather J. M. Reich-Zeliger S. Shifrut E. (2017). Feature Selection Using a One Dimensional Naïve Bayes' Classifier Increases the Accuracy of Support Vector Machine Classification of CDR3 Repertoires. Bioinformatics 33 (7), btw771–955. 10.1093/bioinformatics/btw771 PMC5860388 28073756

[B8] Curatolo P. Moavero R. van Scheppingen J. Aronica E. (2018). mTOR Dysregulation and Tuberous Sclerosis-Related Epilepsy. Expert Rev. Neurotherapeutics 18 (3), 185–201. 10.1080/14737175.2018.1428562 29338461

[B9] Devinsky O. Vezzani A. O'Brien T. J. Jette N. Scheffer I. E. de Curtis M. (2018). Epilepsy. Nat. Rev. Dis. Primers 4, 18024. 10.1038/nrdp.2018.24 29722352

[B10] Fiest K. M. Sauro K. M. Wiebe S. Patten S. B. Kwon C.-S. Dykeman J. (2017). Prevalence and Incidence of Epilepsy. Neurology 88 (3), 296–303. 10.1212/wnl.0000000000003509 27986877 PMC5272794

[B11] Finsterwald C. Fiumelli H. Cardinaux J.-R. Martin J.-L. (2010). Regulation of Dendritic Development by BDNF Requires Activation of CRTC1 by Glutamate. J. Biol. Chem. 285 (37), 28587–28595. 10.1074/jbc.M110.125740 20639200 PMC2937884

[B12] Fisher R. S. Acevedo C. Arzimanoglou A. Bogacz A. Cross J. H. Elger C. E. (2014). ILAE Official Report: a Practical Clinical Definition of Epilepsy. Epilepsia 55 (4), 475–482. 10.1111/epi.12550 24730690

[B13] Friedman J. Hastie T. Tibshirani R. (2010). Regularization Paths for Generalized Linear Models via Coordinate Descent. J. Stat. Softw. 33 (1), 1–22. 10.18637/jss.v033.i01 20808728 PMC2929880

[B14] Guerrini R. Dobyns W. B. (2014). Malformations of Cortical Development: Clinical Features and Genetic Causes. Lancet Neurol. 13 (7), 710–726. 10.1016/s1474-4422(14)70040-7 24932993 PMC5548104

[B15] Harper A. R. Topol E. J. (2012). Pharmacogenomics in Clinical Practice and Drug Development. Nat. Biotechnol. 30 (11), 1117–1124. 10.1038/nbt.2424 23138311 PMC3819119

[B16] Hodges S. L. Lugo J. N. (2020). Therapeutic Role of Targeting mTOR Signaling and Neuroinflammation in Epilepsy. Epilepsy Res. 161, 106282. 10.1016/j.eplepsyres.2020.106282 32036255 PMC9205332

[B17] Khan M. I. Dębski K. J. Dabrowski M. Czarnecka A. M. Szczylik C. (2016). Gene Set Enrichment Analysis and Ingenuity Pathway Analysis of Metastatic clear Cell Renal Cell Carcinoma Cell Line. Am. J. Physiology-Renal Physiol. 311 (2), F424–F436. 10.1152/ajprenal.00138.2016 27279483

[B18] Kuechler A. Buysse K. Clayton-Smith J. Le Caignec C. David A. Engels H. (2011). Five Patients with Novel Overlapping Interstitial Deletions in 8q22.2q22.3. Am. J. Med. Genet. 155 (8), 1857–1864. 10.1002/ajmg.a.34072 21739578

[B19] Kuroda Y. Ohashi I. Saito T. Nagai J.-i. Ida K. Naruto T. (2014). Refinement of the Deletion in 8q22.2-q22.3: the Minimum Deletion Size at 8q22.3 Related to Intellectual Disability and Epilepsy. Am. J. Med. Genet. 164 (8), 2104–2108. 10.1002/ajmg.a.36604 24801133

[B20] Kuzniecky R. (2015). Epilepsy and Malformations of Cortical Development. Curr. Opin. Neurol. 28 (2), 151–157. 10.1097/wco.0000000000000175 25695135

[B21] Kwan P. Arzimanoglou A. Berg A. T. Brodie M. J. Allen Hauser W. Mathern G. (2010). Definition of Drug Resistant Epilepsy: Consensus Proposal by the Ad Hoc Task Force of the ILAE Commission on Therapeutic Strategies. Epilepsia 51 (6), 1069–1077. 10.1111/j.1528-1167.2009.02397.x 19889013

[B22] Kwan P. Brodie M. J. (2000). Early Identification of Refractory Epilepsy. N. Engl. J. Med. 342 (5), 314–319. 10.1056/nejm200002033420503 10660394

[B23] Langfelder P. Horvath S. (2008). WGCNA: an R Package for Weighted Correlation Network Analysis. BMC Bioinformatics 9, 559. 10.1186/1471-2105-9-559 19114008 PMC2631488

[B24] Lerche H. (2020). Drug-resistant Epilepsy - Time to Target Mechanisms. Nat. Rev. Neurol. 16 (11), 595–596. 10.1038/s41582-020-00419-y 33024326

[B25] Li J. Liu C. Chen Y. Gao C. Wang M. Ma X. (2019). Tumor Characterization in Breast Cancer Identifies Immune-Relevant Gene Signatures Associated with Prognosis. Front. Genet. 10, 1119. 10.3389/fgene.2019.01119 31781173 PMC6861325

[B26] Lim J. S. Kim W.-i. Kang H.-C. Kim S. H. Park A. H. Park E. K. (2015). Brain Somatic Mutations in MTOR Cause Focal Cortical Dysplasia Type II Leading to Intractable Epilepsy. Nat. Med. 21 (4), 395–400. 10.1038/nm.3824 25799227

[B27] Liu X. Wang Q. (2015). Screening of Feature Genes in Distinguishing Different Types of Breast Cancer Using Support Vector Machine. Ott 8, 2311–2317. 10.2147/ott.S85271 PMC4556031 26347014

[B28] Löscher W. Potschka H. Sisodiya S. M. Vezzani A. (2020). Drug Resistance in Epilepsy: Clinical Impact, Potential Mechanisms, and New Innovative Treatment Options. Pharmacol. Rev. 72 (3), 606–638. 10.1124/pr.120.019539 32540959 PMC7300324

[B29] Mattson R. H. Cramer J. A. Collins J. F. Smith D. B. Delgado-Escueta A. V. Browne T. R. (1985). Comparison of Carbamazepine, Phenobarbital, Phenytoin, and Primidone in Partial and Secondarily Generalized Tonic-Clonic Seizures. N. Engl. J. Med. 313 (3), 145–151. 10.1056/nejm198507183130303 3925335

[B30] Rawat C. Kushwaha S. Srivastava A. K. Kukreti R. (2020). Peripheral Blood Gene Expression Signatures Associated with Epilepsy and its Etiologic Classification. Genomics 112 (1), 218–224. 10.1016/j.ygeno.2019.01.017 30826443

[B31] Redmond L. Kashani A. H. Ghosh A. (2002). Calcium Regulation of Dendritic Growth via CaM Kinase IV and CREB-Mediated Transcription. Neuron 34 (6), 999–1010. 10.1016/s0896-6273(02)00737-7 12086646

[B32] Rivera J. Proia R. L. Olivera A. (2008). The alliance of Sphingosine-1-Phosphate and its Receptors in Immunity. Nat. Rev. Immunol. 8 (10), 753–763. 10.1038/nri2400 18787560 PMC2600775

[B33] Ryvlin P. Rheims S. Lhatoo S. D. (2019). Risks and Predictive Biomarkers of Sudden Unexpected Death in Epilepsy Patient. Curr. Opin. Neurol. 32 (2), 205–212. 10.1097/wco.0000000000000668 30694923 PMC6779136

[B34] Schmidt D. Schachter S. C. (2014). Drug Treatment of Epilepsy in Adults. Bmj 348, g254. 10.1136/bmj.g254 24583319

[B35] Schwenk R. W. Vogel H. Schürmann A. (2013). Genetic and Epigenetic Control of Metabolic Health. Mol. Metab. 2 (4), 337–347. 10.1016/j.molmet.2013.09.002 24327950 PMC3854991

[B36] Sharma P. Kumar A. Singh D. (2019). Dietary Flavonoids Interaction with CREB-BDNF Pathway: An Unconventional Approach for Comprehensive Management of Epilepsy. Cn 17 (12), 1158–1175. 10.2174/1570159x17666190809165549 PMC7057203 31400269

[B37] Szklarczyk D. Franceschini A. Wyder S. Forslund K. Heller D. Huerta-Cepas J. (2015). STRING V10: Protein-Protein Interaction Networks, Integrated over the Tree of Life. Nucleic Acids Res. 43 (Database issue), D447–D452. 10.1093/nar/gku1003 25352553 PMC4383874

[B38] Thijs R. D. Surges R. O'Brien T. J. Sander J. W. (2019). Epilepsy in Adults. The Lancet 393 (10172), 689–701. 10.1016/s0140-6736(18)32596-0 30686584

[B39] Tomson T. Battino D. Perucca E. (2016). Valproic Acid after Five Decades of Use in Epilepsy: Time to Reconsider the Indications of a Time-Honoured Drug. Lancet Neurol. 15 (2), 210–218. 10.1016/s1474-4422(15)00314-2 26655849

[B40] Wang G. Zhu Z. Xu D. Sun L. (2020). Advances in Understanding CREB Signaling-Mediated Regulation of the Pathogenesis and Progression of Epilepsy. Clin. Neurol. Neurosurg. 196, 106018. 10.1016/j.clineuro.2020.106018 32574967

[B41] Zhang Y. Dong H. Duan L. Yuan G. Liang W. Li Q. (2019). SLC1A2 Mediates Refractory Temporal Lobe Epilepsy with an Initial Precipitating Injury by Targeting the Glutamatergic Synapse Pathway. IUBMB Life 71 (2), 213–222. 10.1002/iub.1956 30360015

[B42] Zhu Y. Elemento O. Pathak J. Wang F. (2019). Drug Knowledge Bases and Their Applications in Biomedical Informatics Research. Brief Bioinform 20 (4), 1308–1321. 10.1093/bib/bbx169 29304188

